# Historical Asbestos Measurements in Denmark—A National Database

**DOI:** 10.3390/ijerph19020643

**Published:** 2022-01-06

**Authors:** Ana Sofia Fonseca, Amalie Kofoed Jørgensen, Bianca Xuan Larsen, Marina Moser-Johansen, Esben Meulengracht Flachs, Niels Erik Ebbehøj, Jakob Hjort Bønløkke, Trine Olesen Østergaard, Jesper Bælum, David Lee Sherson, Vivi Schlünssen, Harald William Meyer, Keld Alstrup Jensen

**Affiliations:** 1 National Research Centre for the Working Environment (NRCWE), 2100 Copenhagen, Denmark; amj@nfa.dk (A.K.J.); bxl@nfa.dk (B.X.L.); mgm@nfa.dk (M.M.-J.); vs@ph.au.dk (V.S.); kaj@nfa.dk (K.A.J.); 2 Department of Occupational and Environmental Medicine, Bispebjerg and Frederiksberg Hospital, University of Copenhagen, 2400 Copenhagen, Denmark; esben.meulengracht.flachs@regionh.dk (E.M.F.); ebbehoj@dadlnet.dk (N.E.E.); harald.william.meyer@regionh.dk (H.W.M.); 3 Department of Occupational and Environmental Medicine, Danish Ramazzini Center, Aalborg University Hospital, 9000 Aalborg, Denmark; jahb@rn.dk (J.H.B.); trineoester10@gmail.com (T.O.Ø.); 4 Occupational and Environmental Medicine Clinic, Department of Pulmonary Medicine, Odense University Hospital, 5000 Odense, Denmark; Jesper.Baelum@rsyd.dk (J.B.); david.sherson@rsyd.dk (D.L.S.); 5 Department of Public Health, Work, Environment and Health, Danish Ramazzini Center, 8200 Aarhus, Denmark

**Keywords:** asbestos fibres, historical exposure measurements, occupational exposure, personal sampling, database, phase contrast microscope

## Abstract

Objectives: Due to the long lag-time for health outcomes, historical asbestos exposure measurements are valuable to support assessments of associated occupational health effects, and also to assess time trends and effects of preventive measures. Methods: Different sources of stored data were collated, assessed and refined to create a harmonized database on historical asbestos fibre concentrations measured in specific work tasks and different industries. The final database contains 9236 asbestos measurements from Danish workplaces collected from 1971 to 1997. Results: The geometric mean of asbestos concentrations in different occupations and tasks ranged from 0.003 to 35 fibres cm^−3^. Highest concentrations were registered during handling of asbestos products in the construction services during the period 1981–1997. Although all the measured asbestos exposures without the use of respiratory equipment by the worker in the period of 1971–1997 exceeded the current 8-h time-weighted average exposure limit of 0.1 fibres cm^−3^, the majority of samples collected in the earlier period of 1971 to 1980 did not exceed the exposure limit of 2 fibres cm^−3^, which was in place at the time. All exposure data obtained from 1980 and onwards were found to be one seventh of the mean fibre concentrations in the previous measurement period. The impact of time shows a clear exponentially decreasing trend-line. Conclusions: Despite limitations in coverage of different occupations and tasks associated with the inventoried historical asbestos measurements, the data are helpful to identify specific work scenarios within an industry, where relatively high asbestos exposure levels may still occur or have occurred from 1971 to 1997.

## 1. Introduction

Asbestos refer to a group of naturally occurring fibrous minerals [1] with different compositions and physical and chemical properties [2]. The fibrous nature of the materials is their common denominator.

Currently, around 125 million people are exposed to asbestos at the workplace worldwide. Asbestos fibres fulfilling the “World Health Organisation (WHO)”-criteria (WHO-criteria: length L > 5 µm, diameter D < 3 µm, aspect ratio L:D > 3:1) are considered hazardous if inhaled or ingested [3,4,5,6,7]. Epidemiologic evidence has shown that all asbestos fibre types cause a wide range of diseases, such as lung, larynx and ovarian cancer, mesothelioma, and asbestosis (an interstitial lung disease) [2,8]. In most cases, more than 20 years pass from the initial asbestos exposure to the occurrence of mesothelioma and lung cancer [9,10]. A dose-response relation is evident between asbestos exposure and the risk of asbestosis, suggesting a 1% lifetime risk of asbestosis at 10 years of fibre exposure; 10 fibre years [11,12].

Since the 19th century, asbestos were widely used in several industries due to its extraordinary tensile strength, and resistance to heat and corrosion [13]. Asbestos can be found worldwide in boilers and heating systems, automotive parts, electrical wires, roofing and flooring materials, adhesives and sealants, insulation products, paints, and other products [10].

In Denmark, the peak consumption of asbestos occurred in the early 1970s, with annual imports of 30,000 tonnes of asbestos. Due to the first asbestos ban on insulation materials in 1972, the import of asbestos slowly began to decrease [14] and rapidly from 1986 when the near-total national asbestos ban was decided [15]. Use of limited quantities in brakes continued until a ban was effective from 2004 [16]. In all uses, chrysotile (CAS number 12001-29-5) is reported to be the most commonly used type of asbestos in Denmark [17,18].

Prior to the bans, approximately 150,000 workers [19] are estimated to have been exposed to asbestos in the Danish construction industry, electricity and heating plants, shipyards, and asbestos cement manufacturing. Asbestos exposure has also occurred in connection with a wide variety of other applications, including insulation of locomotives and train carriages. Despite the national ban [15] and consequently significant reduction in use [17], asbestos-containing materials can still be found in buildings, vessels and ships constructed before 1986. Therefore, overall population can be exposed to asbestos, especially when these products are damaged, or in a state of disrepair. However, building craftsmen and firefighters might be more prone to be exposed to significant levels of asbestos during fire extinguishment and during renovation, repairs, demolition activities or deliberate removal of asbestos-containing materials. Consequently, if proper prevention measures are not taken by the employer, asbestos may pose a health risk to them.

Considering the long latency period of asbestos-related diseases, a significant number of cancers, mesotheliomas, and asbestosis is still expected to occur in Denmark during the next decade due to historical asbestos exposures. In this respect, it is important to note that no threshold has been found for health effects of asbestos exposure for any type of asbestos fibre [20,21]. The occupational exposure limit value set for 8 h time weighted average (8 h-TWA) in Denmark is currently 0.1 fibres cm^−3^ [10], similar to the limit value in European Commission Directive 2009/148/EC of 30 November 2009. In addition, Danish policy requires that occupational exposure is assessed, prevented and employees receive the necessary training and instruction if they work with a material that contains asbestos [10]. Medical monitoring of workers is also required when legal exposure limits and exposure duration are exceeded [10].

To identify whether inhalation exposure levels to asbestos have evolved over time and to describe exposure patterns within relevant industries and occupations, it is necessary to know the historical asbestos exposure concentrations and associated pertinent descriptions of working conditions.

Previously, effort has been made to develop qualitative [22,23,24] and quantitative risk matrices [25] to evaluate asbestos exposures. Subsets of data collations on asbestos exposure have also been made previously, including personal measurements of asbestos from single industries for limited time periods [17,18,26,27,28,29]. However, until now, there has not been any attempt to compile or review the available Danish asbestos measurements in order to characterize the typical range of occupational exposure concentrations to asbestos fibres for tasks performed by different workers over time and within specific industries.

This work aims to bring together existing knowledge, experience and data in regards to past occupational exposure to asbestos in Danish workplaces. A database containing 9236 historical measurements of asbestos concentrations from 1971 to 1997 was developed and analysed. This database is considered very helpful to identify specific work scenarios where relatively high asbestos exposure levels have occurred or may still occur in specific industry branches, especially with building craftsmen during repair, renovation or demolition activities. In this way, the inventoried data can help employers and occupational hygiene professionals to estimate the potential asbestos exposure levels for a process and from this information, support in historical health effect and epidemiological assessments, provide the appropriate risk management measures to control asbestos exposure, and predict potential associated detrimental occupational health effects in the next decade.

## 2. Methodology

### 2.1. Data Collection

The National Database was constructed to provide workers exposure level and associated information on the measurement, contextual information, and exposure history when available. Data were identified and extracted from four different sources which contained asbestos measurement documents made in the period of 1971–1997:

- The National Research Centre for Work Environment (NRCWE) and National archives: 79 asbestos measurements from 1982 to 1986 from various industries collected from [30].
- Aalborg University Hospital: 3068 asbestos measurements from 1971 to 1985 in an asbestos cement factory and in a facility which manufactured insulation materials with and without asbestos.
- Department of Public Health, Aarhus University: 132 measurements of asbestos from 1987 to 1989 during dismantling activities in schools and hospitals.
- Odense University Hospital: 5957 measurements from a facility manufacturing asbestos-containing materials such as transport equipment and friction materials (e.g., brakes) in the period of 1980–1997.

### 2.2. Asbestos Database Structure

The database was structured to provide means for harmonized data collation, and to be suitable for grouping industries and occupations, as well as read-across. Furthermore, it was built to be compatible with the currently available exposure assessment models recommended for conventional chemical substances by Registration, Evaluation, Authorization and Restriction of Chemicals regulations (REACH) in terms of required input parameters [31]. Grouping and read-across rules for inhalation exposure are usually made from mapping the source (or process) information and associated information on exposure levels of a certain chemical substance [32]. The exposure levels and contextual information were extracted from measurement documents by 5 different experts and organized in nine main types of information:

(i). Premises: Filling reference number of the archive, company sector;
(ii). Identification of material: name and CAS number;
(iii). (Industrial sector, job and task codes according to the national classifications, as explained below;
(iv). Purpose of the measurement: either random sampling, sampling due to disease in an employee, sampling requested by the company, regulatory check, research purposes or other;
(v). Determinants—exposure scenario: description of working tasks, environmental conditions such as temperature and humidity, number of people working in the same working area (potentially exposed group of people);
(vi). Determinants—technical conditions and measures to control worker exposure: automation level, processes designed aiming to prevent release, type of local exhaust ventilation, type of general ventilation (mechanical or natural), and personal protective measures;
(vii). Exposure metrics: worker experience in years, exposure duration, exposure frequency;
(viii). Sampling characteristics: sampling position (stationary or personal), original sample track number, anonymous subject identifier, sample date and time, pump flow, sampled volume; sampling and fibres counting method used;
(ix). Exposure data: asbestos concentration in fibres cm^−3^.

The final National database reports information based on the industry type, job code and task code.

Industry codes, based on DSE77 (Statistics Denmark job coding system), were partially available through the industry code (This is only the case for the measurements coming from the ATABAS database. As the measurements in the ATABAS database are from the years 1982–1986, DSE77 codes were used. These are based on the International ISIC Revision2 classification. This classification has been kept in the new database) reported in the original measurements. Furthermore, following the existing classification in the ATABAS database [30], the measurements are classified according to a “job type” code and a “task code” (The “job type” code is based on a coding system applied by the “Arbejdstilsynets Ulykkestatistik” (*translated: The Working Environment Authority Statistics on Accidents*) and “Arbejdstilsynets erhvervssygdomsregister” (*translated: The Working Environment Authority Occupational Disease Register*) at the time of the ATABAS database establishment, while the “task code”, which provide additional detail, was a coding system only used at the Arbejdsmiljøinstituttet, (*translated: Danish Institute of Occupational Health now National Research Centre for the Working Environment*). For measurements, where a detailed contextual description existed, “job codes” and “task codes” were attributed if information had not been recorded previously. 

In total, 9236 records were registered of which 9226 data entries could be linked to 16 specific industry codes, 15 different jobs, and 53 different tasks. 

The inventoried asbestos measurements were grouped into six major occupational categories similarly as in Swuste et al. (2008) [33]: (i) manufacturing of asbestos products; (ii) active handling of asbestos products; (iii) transport, storage, packaging of asbestos products; (iv) maintenance jobs; (v) general supervision of work processes and inspection tasks; and (vi) cleaning activities.

### 2.3. Data Analysis

Even though some of the archives and reports provided general insight about the conditions at the workplaces, most of them lacked essential information regarding the actual asbestos concentrations measured, corresponding work situation (job code), sampled year, exposure controls (e.g., personal respiratory protection equipment (RPE), encapsulation of the process, ventilation systems), and the exact sampling location and duration. Consequently, to minimize risk of erroneous data extractions and interpretations, data were only considered if they, as a minimum, included job code identification, asbestos concentration levels (with and without sampled time), sampling year, and measurement position). After data quality assessments, 5869 out of 9236 measurements were available for further analysis (supplementary information Table S1 and Figure S1). The lack of information on the asbestos concentrations was the main reason to discarding records.

The limited number of available exposure data for specific industries and occupations may result in a risk of bias in the direct interpretation of the historical measurements. However, overall, the data indicate a general trend with higher exposure concentrations back in time. This overall trend was described with a mathematical log-linear gamma model based on a function of the most representative industries and year. In addition, boxplots were created to provide a visual summary of the basic statistics such as median, the dispersion of the data, and signs of skewness. For the comparison of personal asbestos exposure concentrations with health guideline values, basic statistics of geometric mean, minimum and maximum of concentrations in each occupational category for the period 1971–1980 or 1981–1997 were provided along with the information regarding the technical conditions and measures to control worker exposure.

## 3. Results

### 3.1. Measurement of Asbestos Concentrations

A complete National database may be found in the excel file available in the supplementary information (Table S2). The supplementary Table S1, and Figure S1 presents an overview of the 5869 high quality measurements of asbestos exposure in Danish companies for each occupational category, industry and job code by the periods 1971–1980 (2189 measurements, 37%) and 1981–1997 (3680 measurements, 63%). Most measurements were personal (5776 measurements, 98.4%), performed as part of a regulatory check (5722 measurements, 97.5%) and made among automotive and cement industries which manufactured asbestos products (5182 measurements, 88%). In almost all the other occupational categories, purpose of measurement, and of sampling position, the number of available measurements was limited to a few samples (Figures S1 and S2). Detailed information about the type of general ventilation systems in place, control measures, and the use of RPE was unknown in 45–60% of the cases. In 45% of the personal measurements, information lacked on whether the worker used RPE and if the measurements were performed inside or outside the mask (Figure S2).

#### 3.1.1. Manufacturing of Asbestos Products

**Figure 1:**
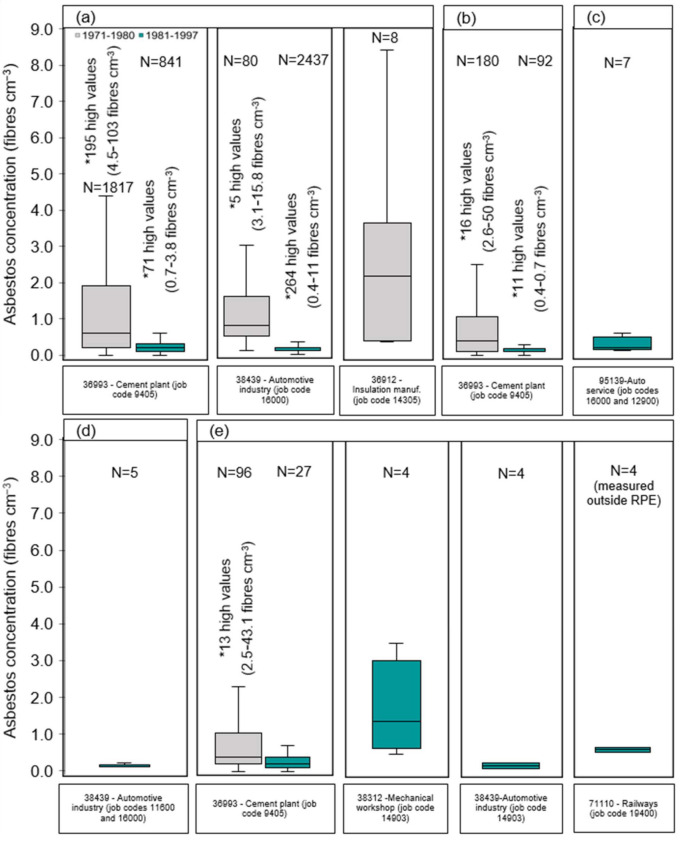
Vertical box plots for the measured personal exposure asbestos concentrations (with N ≥ 4) without respiratory protective equipment being used by the worker (unless specified) for each industry code for the period 1971–1980 and 1981–1997: (**a**) Manufacturing of asbestos products; (**b**) Transport, storage and package; (**c**) Maintenance jobs; (**d**) General supervision and inspection tasks; and (**e**) Cleaning activities. The lower and upper limits of the box plots represent the 25th and 75th percentiles, and the line within the box marks the median. Whiskers (error bars) above and below the box indicate the maximum and the minimum fibre concentration excluding high values (marked as *), respectively. N: total number of measurements available; Manuf: manufacturing; Auto: Automotive.

The manufacturing of fibre cement plates (eternit), insulation plates, and automotive materials (e.g., brakes) in Denmark accounts for 5190 measurements between 1971 and 1997 and involves different activities such as handling of raw materials, cutting and shaping products/materials using manual or power tools, polishing, spraying of asbestos insulation, weighing and mixing of asbestos (Table S1). As illustrated in Figure 1a, median fibre concentrations collected over 6–165 min at the breathing zone of the worker without RPE during the period 1971 to 1980 were found to vary between 0.6 and 2.2 fibres cm^−3^, with maximum registered concentrations levels up to 103 fibres cm^−3^. In the consecutive period 1981–1997, the median exposure decreased considerably, resulting in a total reduction of 79% and 84% in exposure levels during the manufacturing of cement plates and automotive materials, respectively. The use of asbestos in cement products ceased at the end of 1986, and therefore asbestos measurements were not performed after 1985. The reduction in exposure over time during the manufacturing of fibre cement plates can be attributed to the use of mechanical ventilation from 1977 onwards (reported on the original archives). Even though, there is no information regarding the use of control measures during the manufacturing of automotive materials, the reduction of exposure over time can also be explained by the increased enforcement of legal standards from 1980 onwards.

#### 3.1.2. Active Handling of Asbestos Products

**Figure 2:**
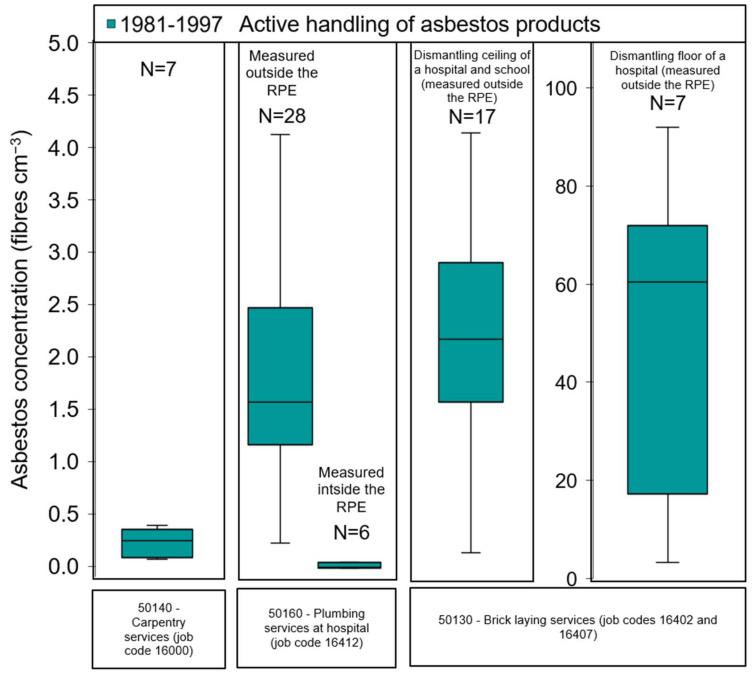
Vertical box plots for the measured personal exposure asbestos concentrations (with N ≥ 4) without respiratory protective equipment being used by the worker (unless specified) during active handling of asbestos products in each industry code for the period 1981–1997. The lower and upper limits of the box plots represent the 25th and 75th percentiles, and the line within the box marks the median. Whiskers (error bars) above and below the box indicate the maximum and the minimum fibre concentration, respectively. N: total number of measurements available.

Handling of asbestos products include 146 measurements in several industrial sectors (e.g., construction, carpentry, railways, power plants, car repair services) during installation, maintenance, renovation, repair or dismantling/demolition of structures with asbestos-containing materials (Table S1 and Figure S1). It is known that high energy tools are often used in these tasks resulting in a high potential of asbestos release and consequently worker exposure. Indeed, Figure 2 and Table S1 show relatively high asbestos exposures levels during activities in building and construction. In 1984, samples collected at the breathing zone of workers without RPE for 15–65 min without any personal protection equipment, fibre concentrations ranged from 0.1 to 4.7 fibres cm^−3^ during building construction and carpentry services, especially during the cutting drilling and band sawing of eternit plates. In the same time period of 1984–1985, 15–77 min personal exposure concentrations without use of RPE were <1.8 fibres cm^−3^ during changing or grinding brakes in the railways or car repair services and assembly of lamp sockets in welfare services.

In Denmark, specialized abatement workers were not common before the adoption of a national ban on asbestos in 1986 [15]. The key tasks involved in abatement work include preparing the work area prior to demolition, and actively removing the asbestos-containing materials. In 1986, the repair of a boiler in a power plant and removal of asbestos-containing insulation materials during 36 min yielded a personal fibre concentration of 4.7 fibres cm^−3^ measured outside the RPE of the worker (Table S1). Similarly, in 1987, personal fibre concentrations measured outside the RPE of the worker were found to range from about 0.24 to 4.1 fibres cm^−3^ during the dismantling of a pipe insulation in a hospital boiler room (Figure 2 and Table S1). During these asbestos measurements, concentrations below 0.06 fibres cm^−3^ were registered inside the RPE of the worker, meaning that the protection efficiency was relatively high and workers exposure were reduced 98.6%. However, if there were workers in the vicinity without RPE, they could have been exposed to fibre concentrations at near field (NF) or far field (FF) in the range of 0.01–2.1 and 0.003–0.01 fibres cm^−3^, respectively (Table S1). The measurements carried out in the decontamination facility around this working area (consisting of 3 different rooms separated by airlocks to prevent the free passage of air or asbestos fibres) revealed that the “clean room” was contaminated throughout the abatement project. In this particular case, stationary concentrations up to 0.02 fibres cm^−3^ were measured. Another observation is that workers who might have spent some time in the contaminated hospital areas before the dismantling activities, were exposed to asbestos concentrations of up to 0.5 fibres cm^−3^ if they had not used RPE (Table S1).

Figure 2 also reveals that in the period of 1986 to 1989, tasks performed by abatement workers during 18–140 min entailed personal exposures measured outside the RPE to asbestos ranging from 0.2 to 4.9 fibres cm^−3^ during removal of asbestos in ceilings in a hospital and elementary school built at early 1970s by using good industrial hygiene practices (encapsulation, use of local exhaust, dilution ventilation and personal protective equipment). These buildings seemed to contain background concentrations of 0.02–0.3 fibres cm^−3^ (25th–75th percentiles) without active contact with the asbestos-containing materials (Table S1). However, after the abatement activities, workers were also exposed to asbestos concentrations ranging from 0.02 to 0.6 fibres cm^−3^ in the clean room (located in the decontamination facility adjacent to the work area).

Much higher personal fibre concentrations, measured outside the RPE, in the range of 3.3–92 fibres cm^−3^ were associated with activities involving removal and scraping of cement floors in a hospital. Simultaneous concentrations measured outside the sanitary area as well as in the clean area of the decontamination facility were 2 orders of magnitude lower (0.01–0.02 fibres cm^−3^). As also indicated in Table S1, in 1983, 1.5 fibres cm^−3^ were measured over 37 min during application of heat to metal pieces (welding) in the civil defence sector by workers who did not use RPE.

#### 3.1.3. Transport, Storage, and Package of Asbestos Products

Warehouse workers could potentially have been exposed during the transport, storage, and packaging of asbestos in bags such as asbestos sheets or wires. This inventory accounts for a total of 363 measurements under these occupational situations. Particularly, in the period 1971–1980, exposure concentrations measured during 37–165 min in an asbestos cement plate factory were 0.1–1.0 fibres cm^−3^ (25th–75th percentiles) with a few extremes varying from 3 to 50 fibres cm^−3^ (Figure 1b and Table S1). Fibre concentrations were found to decrease to 0.1–0.2 fibres cm^−3^ (25th–75th percentiles) when using improved industrial hygiene practices such as the use of mechanical ventilation from 1977 onwards (reported in the original archives). Furthermore, exposure levels found in the automotive industry and civil defence sector for the latest period were <1 fibre cm^−3^ (Table S1).

#### 3.1.4. Maintenance Jobs

Maintenance workers are known to install, test, maintain, and repair electrical wiring, and machinery. This work often involves locating and determining electrical or mechanical failures, testing and adjustments of equipment. Figure 1c illustrates a summary of workers exposure fibre concentrations that have been measured during tasks performed by maintenance workers in automotive services. As indicated in Table S1, 21 measurement samples were found to contain asbestos. In particular, this inventory suggests that electricians and mechanics were exposed to asbestos in 1985–1986 in the range of 0.07 to 0.14 fibres cm^−3^. In addition, in the period of 1983–1985, personal fibre concentrations without use of RPE for most maintenance-related tasks in the clutch and brake services were found to range from 0.1 to 1.5 fibres cm^−3^ based on 15–110 min sampling periods.

#### 3.1.5. General Supervision of Work Processes and Inspection Tasks

This occupational category involves supervision and inspection of places and tasks which can have the potential of asbestos being released to the workplace. This is the case of quality control measurements at the automotive industry to guarantee, that manufacturing of metal products and casting activities are safe to perform and acceptable levels of exposure are ensured. As illustrated in Figure 1d and indicated in Table S1, 6 measurement samples collected at the breathing zone of the worker in 1980–1990 for 45 min were found to contain asbestos in the range of 0.1–1 fibres cm^−3^. Furthermore, 3 samples collected with unknown sampling duration and 1 sample collected during 348 min for the period of 1983–1989 while inspection of places such as schools and regular offices before dismantling of structures containing asbestos resulted in 0.04 to 0.53 cm^−3^ airborne fibre concentrations (Table S1). After dismantling of ceiling in a school, 2 samples collected in the clean room of the decontamination facility revealed that workers might have been exposed to geometric mean asbestos concentrations of 0.02 fibres cm^−3^.

#### 3.1.6. Cleaning Activities

Historical asbestos exposure data found in Danish workplaces also confirmed that several workers were exposed to asbestos during the cleaning of asbestos-containing materials. However these exposure levels were not as high as the manufacturing or active handling of asbestos products on a daily or annual basis. Table S1 and Figure 1e provides a summary of 137 asbestos measurements during cleaning tasks performed by workers in a variety of industries such as the asbestos cement plates manufacturing plant, automotive and construction sectors. Specifically, tasks involving general clean-up at an asbestos cement plant in the period of 1971–1980 were found to result in 0.2–0.95 fibres cm^−3^ (25th–75th percentiles) fibre concentrations collected at the breathing zone of the worker without use of RPE with few extremes reaching up to 43 fibres cm^−3^ based on samples collected for 24–68 min. Fibre concentrations were found to decrease to 0.1–0.35 fibres cm^−3^ (25th–75th percentiles) in the subsequent period of 1981 to 1984 in the same industry sector most likely due to the increased enforcement of legal standards and the use of mechanical ventilation from 1977 onwards. For the time period of 1982–1985, similar exposure levels were found in the automotive industry (0.1–0.3 fibres cm^−3^ (25th–75th percentiles) based on samples collected for 45 min). However, cleaning activities performed in electromechanical workshops, construction sites, and railway services were found to result in higher fibre personal exposure concentrations (measured without or outside the RPE), which ranged from 0.5 to 8.7 fibres cm^−3^ based on samples collected for 5–75 min in the period of 1984–1989, probably due to the confined workplaces and the composition or type of materials being cleaned.

### 3.2. Time Evolution of Exposure Patterns

Prior to 1980, quantitative exposure information was scarce with only 37% of the inventoried situations measured (Supplementary Figure S2). Most quantitative information to describe exposure patterns among industries and jobs was available for the primary asbestos industries which manufactured automotive and cement products corresponding to 96.7% of the total number of measured situations. In almost all the other industries and jobs, the number of available measurements was limited to a few samples. This scattered quantitative exposure information did not allow inference of time trends in the different industries and jobs and, thus, the changes over time were derived from a log-linear gamma model fit applied to the manufacturing industries of asbestos cement plates and automotive components (industry codes 36993 and 39439, respectively). 

**Figure 3:**
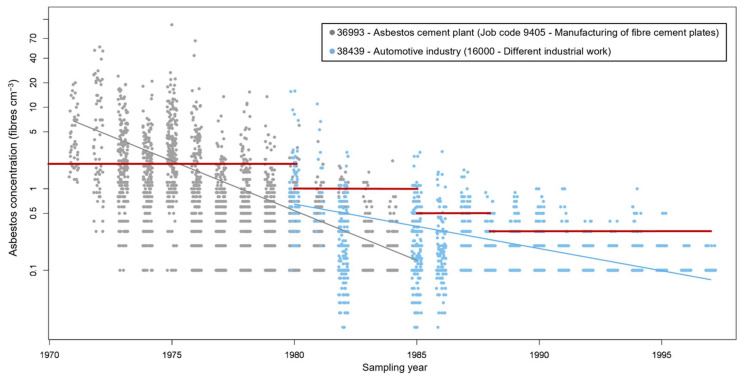
Personal exposure asbestos exposure concentrations against sampled year. The grey and blue lines stand for the log-linear gamma model fit applied for industry sector 36993 and 38439, respectively. All the personal measurements at the asbestos cement plant correspond to exposures without use of RPE, while at the automotive industry it is unknown if RPE was used. Horizontal red lines show the different health guideline values over time. (For interpretation of the references to colour in this figure legend, the reader is referred to the web version of this article).

The impact of time is shown in Figure 3 where concentrations are plotted against year of measurement for these specific industries and corresponding job codes. The obtained yearly exponential decline 0.75 and 0.88, equal to a yearly decrease of 0.25% and 0.12% for industry codes 36,993 and 38,439, respectively. A clear downwards trend, with a somewhat exponential shape is observed with increasing age of measurement (Figure 3). This trend over time seems to reflect the overall development in Danish industries [27,28,29]. Given that the asbestos regulations did not come into force until the 1980s, it is reasonable to assume that occupational exposures to asbestos were generally much higher during the 1970s, as observed, and likely also earlier.

## 4. Discussion

### 4.1. Asbestos Exposure Levels Found in Danish Industries

From the data in the established database on Danish historical asbestos exposures, it was evident that airborne fibre concentrations were highest during active handling of asbestos products in construction services. The highest levels stem from the period 1981–1997, but lack of measurements for this activity before 1981 does not allow us to compare with that period. The asbestos exposure concentrations measured outside the RPE of the worker were found to range from 3.3 to 92 fibres cm^−3^ during removal and scraping of cement floors in a hospital (Table S1 and Figure 2). The Health Effects Institute, United States has also concluded that airborne fibre concentrations during asbestos removal tasks could have been as high as 1 fibre cm^−3^ during wet removal and as high as 10 to 100 fibres cm^−3^ during dry removal [34]. In our data, lower personal exposure concentrations in the range of 0.02 to 4.9 fibres cm^−3^ were measured also outside the RPE of the worker during removal of asbestos in ceilings and during cutting, drilling and band sawing of eternit plates (Table S1 and Figure 2). The measurement report elaborated by the Dutch Labour Inspectorate reported similar ranges of worker concentrations during sawing (1.4–4.3 fibres cm^−3^) and grinding (0.1–0.2 fibres cm^−3^) of water pipes [22].

During the earliest measurement period 1971–1980, manufacturing processes were ranked as being the occupation with the highest exposure concentrations without using RPE (geometric mean: 1.1 to 1.9 fibres cm^−3^ with a few high exposure levels of up to 103 fibres cm^−3^, especially during manufacturing of fibre cement plates, and insulation materials) followed by cleaning of asbestos-containing materials (geometric mean: 0.8 fibres cm^−3^, maximum: 43.1 fibres cm^−3^), and warehouse activities in the asbestos cement industry (geometric mean: 0.7 fibres cm^−3^ with few high exposure levels of up to 50 fibres cm^−3^) (Table S1). According to Raffn et al. (1989) [17] and Raffn (1990) [28], the first measurements of asbestos exposure at a cement manufacturer, were taken in 1949 during asbestos milling activities. The results showed exposure concentrations varying from 85–350 and 150–800 fibres cm^−3^ for 2–15 and 15–200 µm in length, respectively. Follow up measurements in 1957 at the same factory were 10–100 fibres cm^−3^ [29]. These asbestos levels measured before 1971 were not included in the database created in this study because it is unknown how asbestos were measured or estimated in these samples. The levels found during warehouse activities in the asbestos cement industry were slightly higher than the levels found in the Netherlands (from 0.04 to 0.8 fibres cm^−3^) for the same type of activities in the period of 1970–1990 [33]. Several other studies have also reported that tasks involving general cleaning of asbestos-containing materials and debris, were found to result in average fibre concentrations ranging from 0.005 to 4.8 fibres cm^−3^ based on short-term and longer term sampling durations collected at various industrial sites [33,35].

**Figure 4:**
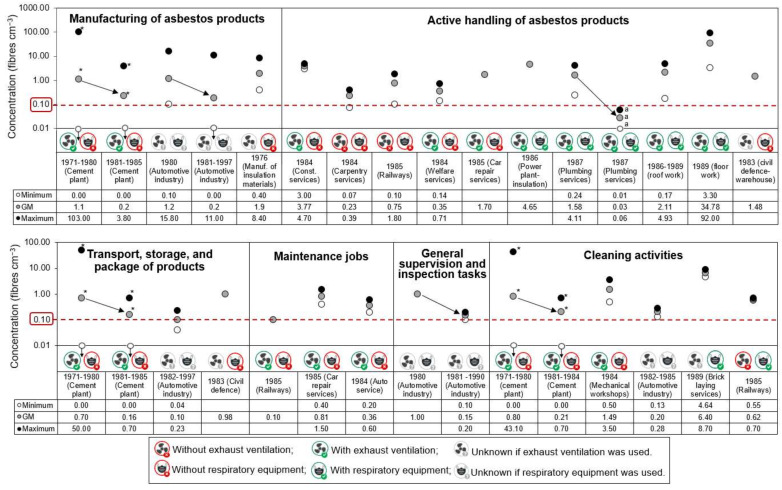
Personal exposure asbestos concentrations in each occupational category for the period 1971–1980 or 1981–1997 compared to the current 8-h TWA health guideline value (horizontal dashed line). The white, grey and black dots correspond to minimum, geometric mean (GM) and maximum asbestos concentration, respectively. Concentrations = 0 fibres cm^−3^ are represented in y = 0.01 with an arrow towards down. All the personal measurements which workers used respiratory protective equipment were taken outside the mask unless specified; *: Mechanical ventilation used from 1977 onwards; ^a^ Measurements taken inside the respiratory protective equipment; Manuf.: manufacturer; Const.: construction.

The inventoried datasets from 1980 onwards were found to have seven-fold reduced geometric mean fibre concentrations during manufacturing processes, cleaning activities, supervision, and warehouse activities in both cement and automotive industries, compared to the earlier measurement periods (Figure 4). The observed exponential decreasing asbestos exposure levels over time towards 1997 are probably explained by: (i) the revision and reduction of the occupational exposure limit set for asbestos; (ii) improved industrial hygiene practices and engineering controls (e.g., enclosure, ventilation, and cleaning procedures); (iii) less exposure time due to the replacement of certain asbestos-containing products; and (iv) increased awareness of occupational hazards, health problems and safety measures. Lower exposure levels with time is in agreement with results in Swuste et al. (2008) [33] who also reported a drop in exposure levels to asbestos during manufacturing of cement products in the Netherlands ranging from 0.9–2.7 fibres cm^−3^ during the period 1970–1979 and 0.09–0.12 fibres cm^−3^ in the period 1980–1989. Similarly, measured fibre concentrations were found to range from 0.01 to 2.4 fibres cm^−3^ during production friction materials during the period of 1970 to 1984 in the Netherlands [22,36], and varying from 1.2–12 fibres cm^−3^ during production of brake linings before 1974 in Sweden [37]. 

The lowest personal exposure concentrations without the use of RPE were observed for maintenance workers (geometric mean <0.8 fibres cm^−3^) and workers with indirect contact during 1980–1990, primarily involved in process general supervision and inspection tasks during manufacturing (geometric mean <1 fibres cm^−3^) or in activities that do not require manipulation of products (e.g., office work <0.04 fibres cm^−3^) (Table S1 and Figure 4). These exposure levels are quite well in line with the average exposure of 0.2 fibres cm^−3^ found in in the Netherlands during supervision and inspection in the asbestos cement industry for the period of 1975–1979 [33]. Other studies also reported that most maintenance-related tasks made by electricians and mechanics resulted in arithmetic mean fibre concentrations ranging from about <0.01 to 1 fibres cm^−3^ [35,38,39]. The review study conducted by the Health Effects Institute concluded that airborne fibre concentrations during maintenance tasks in public and commercial buildings could have been kept below 0.1 fibres cm^−3^ with proper controls, but could have exceeded 10 fibres cm^−3^ during some removal and repair work without adequate controls in place [34].

Even though only 55% of the measurements reported the use of RPE and sampled location, i.e., outside or inside the RPE, we can conclude that adoption of this control measure increased after 1986 and it became prevalent in construction and automotive industries in work scenarios such as removal of asbestos-containing materials and cleaning activities which yielded the highest geometric mean personal fibre concentrations measured outside the RPE of the worker in the range of 0.6–35 fibres cm^−3^ (Figure 4). Considering the protection efficiency of the RPE, it is reasonable to assume that workers were exposed to significantly lower levels of asbestos than measured (see the demonstrative case of plumbing services in Figure 4 showing that average exposure levels measured inside the RPE were reduced 58 times, corresponding to 98.3% reduction)

### 4.2. Comparison of Asbestos Exposure Concentrations with Health Guideline Values

In the 1970s, the Danish Working Environment Authority set the first health guideline value (HGV) for asbestos expressed as 8 h-TWA of 2 fibres cm^−3^ [40]. Between 1980 and 1985, the exposure concentrations should not exceed the HGV for 8 h-TWA and short term-15 min of 1 and 5 fibres cm^−3^, respectively. From the time period 1985–1988 and 1988–2005, the HGV for 8 h-TWA was reduced from 0.5 to 0.3 fibres cm^−3^ [40]. Since 2005, the Danish HGV for asbestos is 0.1 fibres cm^−3^ for 8 h-TWA and 1 fibre cm^−3^ for short term 15 min exposure [10].

From the inventoried asbestos exposure data (Table S1), nearly all measurements were collected during specific short duration job tasks varying from 6 to 165 min. Because the sampling durations did not match either the 8-h TWA or the short term-15 min HGV, it is challenging to evaluate the inventoried data regarding specific limit values. Since most of the short-term samplings were performed as part of a regulatory check, it is very likely that they correspond to dustiest tasks in a specific industry and would result in an upward bias. On the other hand, it is plausible that greater care was taken to avoid excess dust production and to work by the rules on days of regulatory measurements at the industrial facilities investigated, resulting in an underestimation of the exposure concentrations. Therefore, only rough comparisons can be made by assuming that the average personal air sampling data collected for each industry code in different occupational categories were representative of 8-h TWA measurements.

Even though geometric mean personal fibre exposure concentrations obtained in all of the occupational situations measured without or outside the RPE from 1971 to 1997, exceeded the current HGV of 0.1 fibres cm^−3^ for 8 h-TWA (Figure 4), the majority of samples collected in the period of 1971 to 1980 during manufacturing of automotive components and general supervision and inspection tasks were below the HGV of 2 fibres cm^−3^. Assuming that the use of a RPE reduce 98.3% of the airborne levels, it is likely that geometric mean of workers exposure did not exceed the current HGV of 0.1 fibres cm^−3^ for 8 h-TWA on the exposure scenarios, during where workers used RPE, except during activities involving removal and scraping of cement floors in a hospital which might have exceeded 6-fold (Figure 4).

### 4.3. Database Limitations

The data in the Danish Asbestos Exposure Database have some limitations which affect the ability to compare data and interpret the historical measurement data:

- Most of the inventoried datasets did not provide comprehensive details with respect to their study approach or design to be able to fully understand the type or duration of exposure. Even though some of the archives and reports provided general insight about the working conditions in the workplaces, most of them lacked essential information regarding the corresponding industry, work situation, determinants of exposure (e.g., personal RPE, encapsulation of the process, exhaust ventilation), and duration of measurements. From several exposure scenarios, it was unclear whether the data was based on stationary or personal sampling and sampling year was not reported. In case of personal sampling, it was also not stated in 44.6% of the cases whether such measurement was with RPE and if it was measured inside or outside the respiratory mask. Prior to 1980, exposure scenarios were scarce with only 2189 measurements, corresponding to 37% of the inventoried situations measured in which asbestos concentrations, sampling position, industry and job codes were available (5869 measurements). The lack of these type of descriptions and the scattered quantitative exposure information over time consequently introduced uncertainties in the identification and modelling of clear temporal trends in asbestos exposure levels due to changes in the measurement strategy, exposure control practices and process characteristics [33,41,42].
- Even though, it is very likely that majority of the inventoried asbestos exposure concentrations were determined by using appropriate methodologies, and analysed by using phase contrast microscope (PCM) method, 5817 (out of 5869) datasets lacked information about sampling techniques and counting procedures. One of the PCM method limitations is that it cannot differentiate between asbestos and non-asbestos fibres, while scanning electron microscope (SEM) or transmission electron microscope (TEM), which is approximately 100 times more sensitive, is capable of distinguishing different fibre types. It is therefore likely that PCM method overestimated the asbestos fibre concentration in the air in occupational settings where large proportions of other fibres (e.g., wool, cotton, glass) are present [43,44]. On the other hand, due to the low resolution of the PCM method, it is also probable that most of samples did not account for thin fibres (width less than 0.25 µm) and potentially underestimated asbestos exposures [24,45]. There have been, however, numerous attempts to convert total fibre counts to specific fibre counts with fibre type, length, and diameter [24].
- Most quantitative information describing exposure patterns among industries and jobs was available for the largest Danish industries which manufactured automotive and asbestos cement products (5677 measurements, 96.7%). All the asbestos samplings in these industries were conducted as part of a regulatory check. It is likely that they correspond to the dustiest tasks and would result in an overestimation of personal exposure concentrations if applied to the entire work force in these industries. The detailed information about tasks performed does, however, allow for estimation of the exposure associated with these tasks, which would most likely be close to the typical exposure levels between 1971 and 1985. In almost all the other occupational categories and industries, the number of available measurements was limited to a few samples. It is probable that larger industries registered lower fibre levels if compared to smaller workplaces or less controlled facilities where the awareness of occupational hazards and health problems may have been lower and where safety measures were less sophisticated or even non-existent. The measurements on an asbestos cement factory operating in Denmark were performed quarterly by the factory, systematically covering relevant tasks and analysed at NRCWE (at the time called SIFA) in agreement with the Danish Working Authorities. The insulation materials manufacturer, by far the largest facility of its type in Denmark, performed measurements in a similar way. In principle, these datasets should be highly representative for these workplaces, but evidently, these companies would have been able to affect the results by optimizing tasks, cleaning and ventilation settings on days of measurements. However, the authors believe that if further asbestos exposure concentrations exist, inclusion of these would not significantly affect the key findings reported here.

## 5. Conclusions

This work presents the first known attempt to compile available asbestos measurements in Danish workplaces in order to characterize the typical airborne fibre concentration ranges associated with contextual information for specific work tasks involved in different industries over time. The historical asbestos exposure database contained 9236 records of which 5869 data entries contained high quality measurements of asbestos concentrations from 1971 to 1997.

The highest airborne fibre concentrations were registered during the active handling of asbestos products in construction services during the period 1984–1989. The exposures in the construction industry can in particular be linked to the removal of asbestos-containing floors, and ceilings. For the period 1971–1980, manufacturing processes of eternit cement plates, brake pads and insulation materials were ranked as the occupations with the highest exposure concentrations followed by cleaning of asbestos-containing materials, and warehouse activities. The lowest personal exposure concentrations were observed in the period of 1980–1990 for maintenance workers and workers involved in processes of general supervision and inspection tasks or in activities that do not require manipulation of products.

All of the occupational situations measured without the use or outside the RPE in the period of 1971–1997 exceeded the current HGV of 0.1 fibres cm^−3^ for 8 h-TWA (sometimes by 100-fold or more). However, the majority of samples collected for less than 8 h in the period of 1971 to 1980 were below the contemporaneous HGV of 2 fibres cm^−3^. If a RPE with a filtration efficiency of at least 98.3% would be used in all the inventoried scenarios, the average workers exposure would be below the HGV of 0.1 fibres cm^−3^ for 8 h-TWA except for activities involving removal and scraping of cement floors in a hospital. 

The applied mathematical log-linear gamma model to the manufacturing industries of asbestos cement plates and automotive components confirms a clear downwards trend over time. Given the ban on the use of asbestos in 1986, and the regulatory enforcement on reducing asbestos exposure in the period 1980–2005, it is reasonable to assume that occupational asbestos exposures were generally higher during the 1970s and earlier.

Even though the database on historical asbestos measurements presented in this study have some limitations, the data allowed the identification of specific work scenarios where relatively high asbestos exposure levels occurred and might still occur in the construction sector during repair, renovation or demolition activities. In this way the data can support in historical health effect assessment and epidemiological assessments, but also provide relevant information for current risk assessment, management procedures and prediction of potential associated detrimental occupational health effects in the next decade.

## Data Availability

The data presented in this study are available in Table S2.

## Supplementary Materials

The following supporting information can be downloaded at: https://www.mdpi.com/article/10.3390/ijerph19020643/s1. Figure S1. Overview of the 5869 high quality measurements of asbestos exposure in Danish companies distributed among: (a) all the occupational categories and each of the occupational category and industry code by the periods 1971–1980: (b) manufacturing of asbestos products; (c) active handling of asbestos products; (d) transport, storage, packaging of asbestos products; (e) maintenance jobs; (f) general supervision of work processes and inspection tasks; and (g) cleaning activities. N: total number of measurements available. Figure S2. Overview of the 5869 high quality measurements of asbestos exposure in Danish companies distributed among the following exposure determinants: (a) sampled year; (b) purpose of the measurement; (c) sampling position; (d) type of general ventilation system; (e) control measures in place; and (f) use of respiratory protective equipment (RPE). N/A: not available information; * unknown if RPE was used and unknown if measurement position was located inside or outside the RPE. Table S1. Simplified structure of the Danish asbestos database containing concentration of asbestos fibres collected on filters during specific work shifts, date periods (1971–1980 or 1981–1997), and measurement positions. GSD: stands for the geometric standard deviation. NF: Near field; FF: Far field; N/A: Not available data; N/R: Not recorded; -: Not applicable; RPE: respiratory protection equipment; LEV: Local exhaust ventilation; MV: Mechanical ventilation; Personal*: unknown if measurement position was located inside or outside the RPE; ¥: Used mechanical ventilation from 1977 onwards. Table S2. Historical asbestos measurements in Denmark—National database.
