# Supplementary material for: Historical Asbestos Measurements in Denmark—A National Database
Source: Int J Environ Res Public Health. 2022 Jan 6;19(2):643. doi: 10.3390/ijerph19020643 (PMC8775413; doi:10.3390/ijerph19020643)
Supplement: Supplementary file 1 [file ijerph-19-00643-s001.zip › Supplementary materials_final_cleaned.pdf]

Historical asbestos measurements in Denmark - a National Database

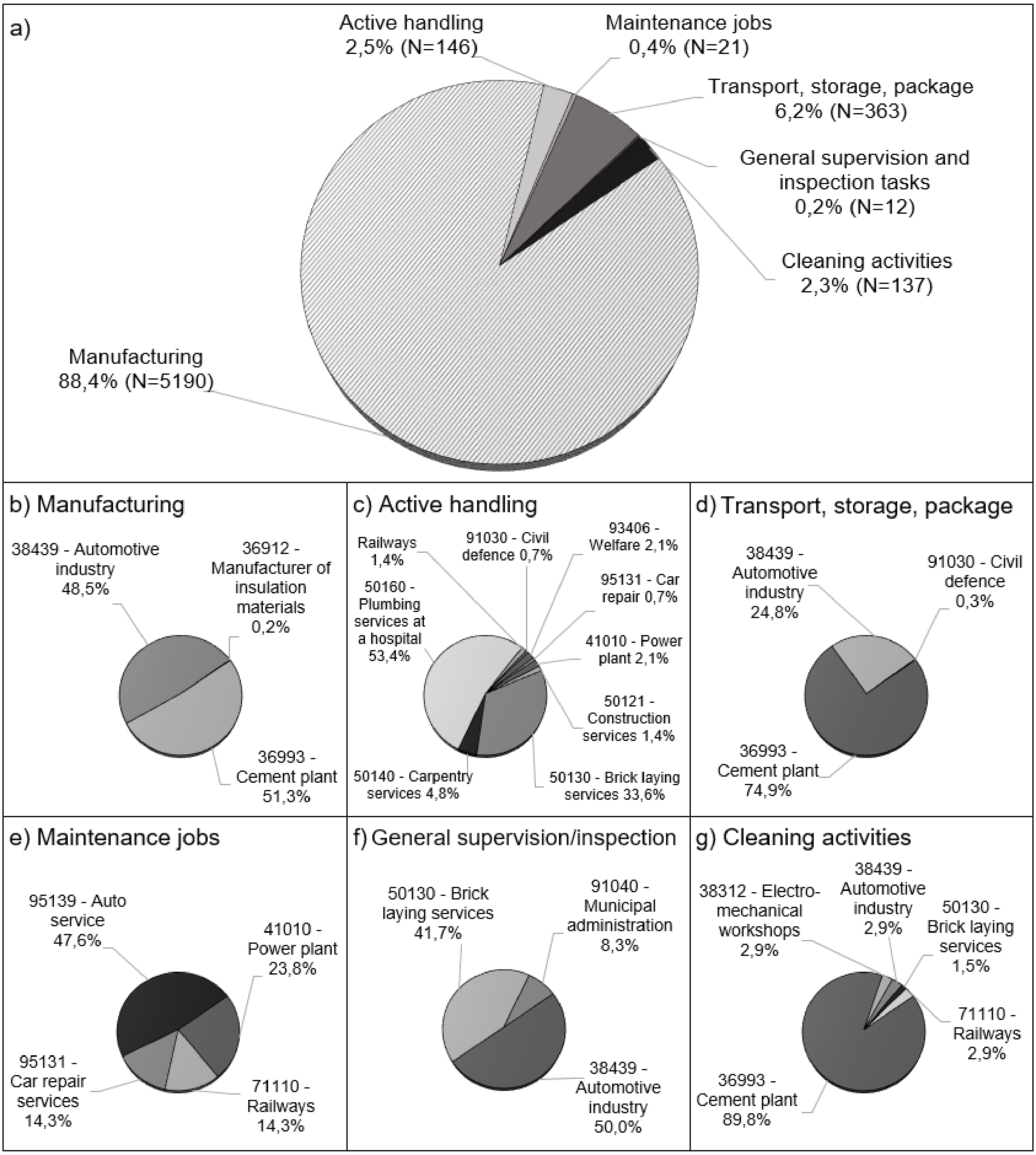

Figure S1. Overview of the 5869 high quality measurements of asbestos exposure in Danish companies distributed among: a) all the occupational categories and each of the occupational category and industry code by the periods 1971-1980: b) manufacturing of asbestos products; c) active handling of asbestos products; d) transport, storage, packaging of asbestos products; e) maintenance jobs; f) general supervision of work processes and inspection tasks; and g) cleaning activities. N: total number of measurements available.

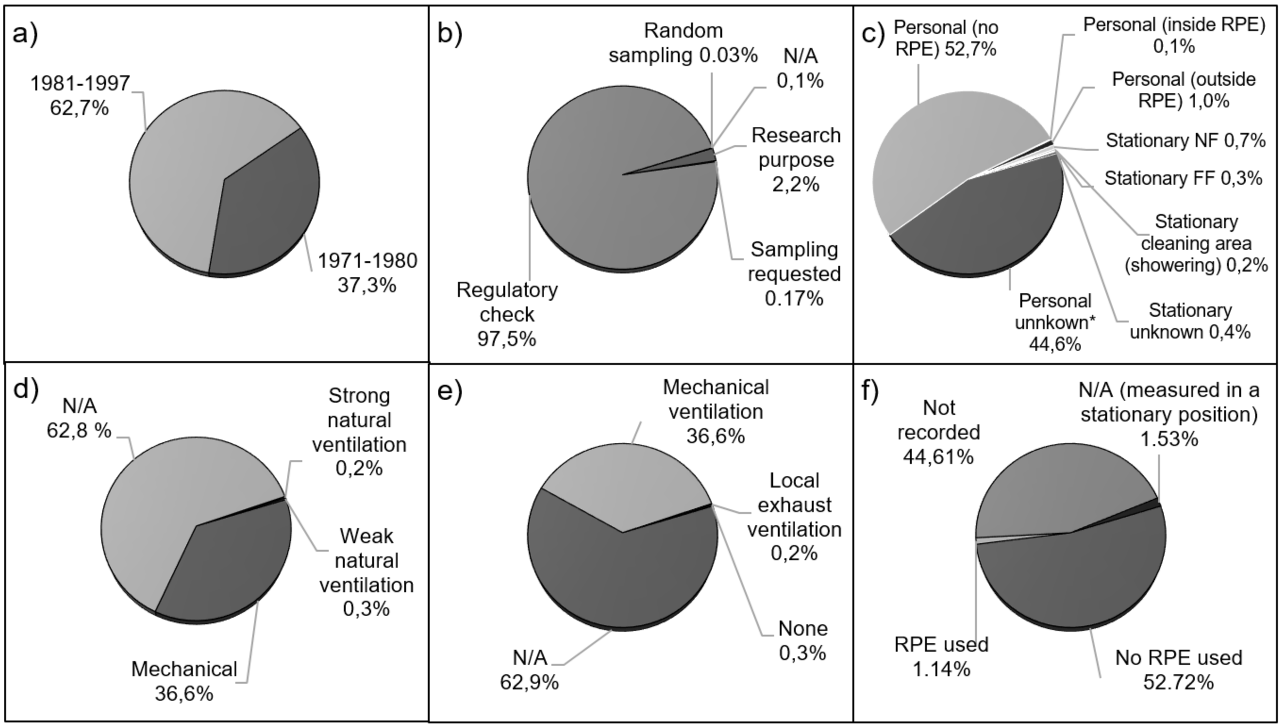

Figure S2. Overview of the 5869 high quality measurements of asbestos exposure in Danish companies distributed among the following exposure determinants: a) sampled year; b) purpose of the measurement; c) sampling position; d) type of general ventilation system; e) control measures in place; and f) use of respiratory protective equipment (RPE). N/A: not available information; *unknown if RPE was used and unknown if measurement position was located inside or outside the RPE.

Table S1. Simplified structure of the Danish asbestos database containing concentration of asbestos fibres collected on filters during specific work shifts, date periods (1971-1980 or 1981-1997), and measurement positions. GSD: stands for the geometric standard deviation. NF: Near field; FF: Far field; N/A: Not available data; N/R: Not recorded; -: Not applicable; RPE: respiratory protection equipment; LEV: Local exhaust ventilation; MV: Mechanical ventilation; Personal: unknown if measurement position was located inside or outside the RPE; ¥: Used mechanical ventilation from 1977 onwards.*

Occupational category	Industry code*	Job code	Period date	Number of measurements with concentrations provided	Sample duration	Measurement position	Purpose of the measurement	Use of control measures	Use of RPE	Geometric mean concentration (fibre cm^{-3})	±GSD	Minimum (fibre cm^{-3})	Maximum (fibre cm^{-3})
**Manufacturing asbestos products**	36993 - Asbestos cement plant	9405 - Manufacturing of fibre cement plates (eternit)	1971-1980	1817 (only 132 with sample duration)	6-165 min	Personal	Regulatory check	Yes¥	No	1.1	1.0	0	103
			1975-1976	6 (only 1 with sample duration)	45 min	Stationary NF	Regulatory check	N/A	-	2.4	0.7	1.7	5.8
			1971	1	99 min	Stationary unknown location	Regulatory check	N/A	-	6.0	-	0.9	6
			1981-1985	841 (only 38 with sample duration)	34-135 min	Personal	Regulatory check	Yes¥	No	0.2	0.2	0	3.8
	36912 - Manufacturer of insulation materials	14305 - Manufacturing of insulation materials	1976	8	N/A	Personal (no RPE)	Regulatory check	N/A	No	1.9	1.0	0.4	8.4
	38439 - Automotive industry	16000 - Different industrial work	1980	80	45 min	Personal*	Regulatory check	N/A	N/R*	1.2	0.7	0.1	15.8
			1981-1997	2437	45 min	Personal*	Regulatory check	N/A	N/R*	0.2	0.2	0	11
**Active handling of**	50121 - Construction services	16000 - Different	1984	2	15 min	Personal (no RPE)	N/A	Yes (LEV)	No	3.77	0.19	3	4.7

**asbestos products**	50140 - Carpentry services	industrial work	1984	7	61-65 min	Personal (no RPE)	Regulatory check	No	No	0.23	0.10	0.07	0.39
	41010 - Power plant		1983	1	96 min	Stationary unknown location	Sampling requested	Yes (LEV)	-	0.70	-	0.7	0.7
	71110 - Railways		1985	2	29-44 min	Personal (no RPE)	Regulatory check	No	No	0.75	0.60	0.1	1.8
	93406 - Welfare services		1984	3	15-77 min	Personal (no RPE)	Sampling requested	Yes (MV)	No	0.35	0.19	0.14	0.71
	95131 - Car repair services		1985	1	15 min	Personal (no RPE)	Regulatory check	Yes (MV)	No	1.70	-	1.7	1.7
	41010 - Power plant		1986	1	47 min	Stationary unknown location	Regulatory check	Yes (MV)	-	0.07	-	0.07	0.07
			1986	1	36 min	Personal (outside RPE)	Regulatory check	Yes (MV)	Yes	4.65	-	4.65	4.65
	50160 - Plumbing installers, and gas and oil boiler service in a hospital	16412 - Insulation work, pipes and containers only	1987	2	N/A	Personal before dismantlin g (no RPE)	Research purpose	yes (MV)	No	0.27	0.03	0.24	0.31
			1987	28	N/A	Personal (outside RPE)	Research purpose	yes (MV)	Yes	1.58	0.46	0.24	4.11
			1987	6	N/A	Personal (inside RPE)	Research purpose	yes (MV)	Yes	0.03	0.02	0.01	0.06
			1987	3	N/A	Stationary FF (before dismantlin g)	Research purpose	yes (MV)	-	0.01	0.001	0.01	0.01
			1987	5	N/A	Stationary FF	Research purpose	yes (MV)	-	0.01	0.00	0.003	0.01

		1987	3	N/A	Stationary NF (before dismantling)	Research purpose	yes (MV)	-	0.31	0.108	0.16	0.49
		1987	21	N/A	Stationary NF	Research purpose	yes (MV)	-	0.52	0.36	0.01	2.05
		1987	5	N/A	Stationary cleaning area (before dismantling)	Research purpose	yes (MV)	-	0.003	0.006	0	0.02
		1987	1	N/A	Stationary cleaning area (after dismantling)	Research purpose	yes (MV)	-	0.01	-	0.02	0.02
		1987	4	N/A	Stationary unknown location (before dismantling)	Research purpose	yes (MV)	-	0.01	0.01	0	0.02
50130 - Brick laying services	19101 - Building work in a hospital	1986	1	265 min	Stationary unknown location	Random sampling	No	-	0.11	-	0.11	0.11
	16402 - Roofing work	1986-1989	17 (only 7 with sample duration)	18-140 min	Personal (outside RPE)	Research purpose (16) and Random sampling (1)	Yes-MV (11) and N/A(6)	Yes	2.1	0.5	0.17	4.93
			8	N/A	Stationary NF	Research purpose	Yes-MV (5) and N/A (3)	-	1.10	0.32	0.47	2.21

				5	N/A	Stationary FF	Research purpose	N/A	-	0.03	0.01	0.02	0.06
				3	N/A	Stationary cleaning area (after dismantling)	Research purpose	N/A	-	0.33	0.21	0.02	0.57
		16407 - Floor covering work of a hospital	1989	7	N/A	Personal (outside RPE)	Research purpose	Yes-MV (4) and N/A (3)	Yes	34.8	1.71	3.3	92
			1989	4	N/A	Stationary NF	Research purpose	Yes-MV (2) and N/A (2)	-	3.43	1.14	0.72	9.4
			1989	2	N/A	Stationary FF	Research purpose	N/A	-	0.02	0.01	0.01	0.02
		18902 – Building renovation in a school	1989	2	N/A	Personal cleaning area (inside RPE)	Research purpose	N/A	Yes	0.01	0.01	0.004	0.02
	91030 - Defence and civil defence	28300 - Warehouse work	1983	1	37 min	Personal (no RPE)	Sampling requested	N/A	No	1.48	-	1.48	1.48
**Transport, storage, and package of asbestos products**	36993 – Asbestos cement plant	9405 - Manufacturing of fibre cement plates (eternit)	1971-1980	180 (only 12 with sample duration)	37-165	Personal	Regulatory check	Yes¥	No	0.70	0.81	0	50
			1981-1985	92	N/A	Personal	Regulatory check	Yes¥	No	0.16	0.12	0	0.7
	38439 - Automotive industry	16000 - Different industrial work	1982-1997	90	45 min	Personal*	Regulatory check	N/A	N/R*	0.10	0.02	0.04	0.23

	91030 - Defence and civil defence	28300 - Warehouse work	1983	1	37 min	Personal (no RPE)	Sampling requested	N/A	No	0.98	-	0.98	0.98
**Maintenance jobs**	41010 - Power plant	15101 - Electricity work	1986	5	169-179 min	Stationary unknown location	Regulatory check	Yes (MV)	-	0.11	0.02	0.07	0.14
	71110 - Railways	16000 - Different industrial work	1985	1	76 min	Personal (no RPE)	Regulatory check	Yes (MV)	No	0.10	-	0.1	0.1
			1985	2	95-96 min	Stationary unknown location	Regulatory check	Yes (MV)	-	0.0	0.00	0	0
	95131 - Car repair services	12900 - Processing of iron and other metals	1985	3	15-27 min	Personal (no RPE)	Regulatory check	Yes (MV)	No	0.81	0.27	0.4	1.5
	95139 - Auto service	16000 - Different industrial work	1983-1985	3	23-75 min	Personal (no RPE; 2) and personal*	Regulatory check (2) and N/A (1)	Yes – LEV(1), No (1), and N/A (1)	No (2) and N/R* (1)	0.16	0.03	0.12	0.2
		12900 - Processing of iron and other metals	1984	4	15-110 min	Personal (no RPE)	Regulatory check	Yes (LEV)	No	0.36	0.14	0.2	0.6
			1984	3	15-85 min	Stationary unknown location	Regulatory check	Yes (LEV)	-	0.75	0.92	0.1	3.4
**General supervision of work processes and inspection tasks**	38439 - Automotive industry	11600 - Manufacturing of metal products and casting	1980	1	45 min	Personal*	Regulatory check	N/A	N/R*	1.00	-	1	1
			1981	2	45 min	Personal*	Regulatory check	N/A	N/R*	0.1	0.044	0.1	0.2
		16000 - Different	1989-1990	3	45 min	Personal*	Regulatory check	N/A	N/R*	0.10	0.00	0.1	0.1

		industrial work											
	50130 - Brick laying services	18902 - Building renovation in a school	1989	2	N/A	Stationary FF (before dismantling)	Research purpose	N/A	-	0.1	0.00	0.11	0.11
			1989	1	N/A	Stationary NF (before dismantling)	Research purpose	N/A	-	0.53	-	0.53	0.53
			1989	2	N/A	Stationary cleaning area (after dismantling)	Research purpose	N/A	-	0.02	0.0005	0.02	0.02
	91040 - County municipal administration	20100 - Office work	1983	1	348 min	Stationary NF (before renovation)	Regulatory check	Yes (MV)	-	0.04	-	0.04	0.04
**Cleaning activities**	36993 – Asbestos cement plant	9405 - Manufacturing of fibre cement plates (eternit)	1971-1980	96 (only 2 with sample duration)	24-68 min	Personal	Regulatory check	Yes¥	No	0.80	1.03	0	43.1
			1981-1984	27	N/A	Personal	Regulatory check	Yes¥	No	0.21	0.15	0	0.7
	38312 - Electromechanical workshops	14903 - Cleaning, miscellaneous	1984	4	5-40 min	Personal (no RPE)	Regulatory check	Yes (MV)	No	1.49	0.48	0.5	3.5
	38439 - Automotive industry		1982-1985	4	45 min	Personal*	Regulatory check	N/A	N/R*	0.20	0.06	0.13	0.28
	50130 - Brick laying services	19400 - Cleaning work	1989	2	N/A	Personal (outside RPE)	Research purpose	N/A	Yes	6.40	0.31	4.64	8.7

71110 Railways	-	1985	4	75 min	Personal (outside RPE)	Sampling requested	No	Yes	0.62	0.04	0.55	0.7

*Danmarks Statistiks Erhvervsgrupperingskode 1977 (DSE77), v5:198
